# Assessment of knowledge on abortion law and factors affecting it among regular undergraduate female students of Ambo University, Oromia Region, Ethiopia, 2018: a cross sectional study

**DOI:** 10.1186/s40834-020-00136-3

**Published:** 2020-12-01

**Authors:** Mulugeta Mekuria, Dechasa Daba, Teka Girma, Adamu Birhanu

**Affiliations:** 1 Department of Public health, Ambo University College of Medicine and Health Science, Po Box 19, Ambo, Ethiopia; 2 West Shoa Health office, West Shewa, Ethiopia; 3 Department of Psychiatry, Ambo University College of Medicine and Health Science, Ambo, Ethiopia

**Keywords:** Abortion, Ambo, Female, Knowledge, Law, Students, University

## Abstract

**Background:**

Knowledge of abortion law is a key determinant of the utilization of safe abortion services. Neglecting it can lead to high social and economic costs, both immediately and in the future. One of the major reasons for not utilizing the safe abortion by the youth female is inadequate knowledge about the abortion law. Therefore the aim of this study was to assess knowledge on abortion law and associated factors among female regular undergraduate students of Ambo University.

**Methodology:**

Institutional-based cross-sectional study was conducted on 795 randomly selected undergraduate female students of Ambo University using structured and pre-tested questionnaire from March, 28-May, 18, 2018.The result of the analysis was presented by tables using descriptive frequency percentage and odd ratios. The result was presented using tables. Bivariate and multivariate logistic regression was done between dependent and independent variables at 95% confident interval and *P* value < 0.05.

**Result:**

Majority (79%) of the study participants were not able to identify all the conditions under which abortion is legally available in Ethiopia. In this study receiving health education on abortion law (AOR = 7.382, 95% CI: (1.118–48.761), knowing where safe abortion can be performed (AOR = 3.116, 95% CI: (1.854–5.239)) and being member of health club in the university (AOR = 2.092, 95% CI:1.256–3.484) were the predictors of abortion law knowlwdge .

**Conclusion:**

The study concluded that knowledge of female student’s on abortion law is low in the study area. In this study, participates who received health education on abortion law and having involving in the health club at university were found to be the predictors of knowledge on abortion law .

## Background

Abortion is intentional or unintentional termination of pregnancy [1] Worldwide about 205 million female become pregnant annually, and an estimated 40–50 million women undergo abortions, 20 million of which are considered unsafe and of all unsafe abortions 95% take place in developing countries From this 43% occurred in Africa and this accounts for an estimated 14% of maternal death in the region [2]**.** In most African countries abortion has been illegal and associated with unsafe abortion and high maternal morbidity and mortality rate [3]. In developed countries the recent policy reforms towards abortion law have reduced the problem associated with illegal and usafe abortion significantly [4]. Some Sub-Saharan African countries including Ethiopia have formulated abortion laws that allow safe abortion [5]. This law states that abortion can be performed legally based on woman’s request in cases of rape or incest, if the woman has physical or mental disabilities, if there is serious fetal deformity and observed needed to preserve the woman’s life or health, or if the pregnant girl is physically or mentally unprepared for childbearing because of her age [6]. Lack of awareness of this abortion law forces female youths to seek unsafe abortion in a secret place [7]. Research in several countries found that public knowledge about abortion law is often minimal [8–10]. The Ethiopian case is not different from other countries and that is why this study was conducted to assess the knowledge of regular female student on the abortion law and factors affecting. Abortion laws have a spectrum of restrictiveness. Evidence indicates that there is an association between unsafe abortion and restrictive abortion laws. The median rate of unsafe abortions in the 82 countries with the most restrictive abortion laws is up to 23 of 1000 women compared with 2 of 1000 in nations that allow abortions [11]. The impact of abortion law on women’s health and survival is high and abortion related deaths are more frequent in countries with more restrictive abortion laws [12].

The same correlation appears when a given country tightens or relaxes its abortion law, for example in Romania where abortion was available upon request until 1966; the abortion mortality ratio was 20 per 100,000 live births in 1960. But when new legal restrictions were imposed in 1966, and by 1989 the ratio reached 148 deaths per 100,000 live births. The restrictions were reversed in 1989, and within a year the ratio dropped to 68 of 100,000 live births; by 2002 it was as low as 9 deaths per 100,000 births [13]. Similarly, in South Africa, after abortion became legal and avail available on request in 1997, abortion related infection decreased by 52%, and the abortion mortality ratio from 1998 to 2001 dropped by 91% from its 1994 level [14]..

One of the primary methods for preventing unsafe abortion is providing less restrictive abortion laws particularly in developing nations, where most unsafe abortions (97%) occur. Less restrictive abortion laws do not appear to entail more abortions overall. Even where these laws were availed by the government to its people, women s need to be educated and a warred about the availability of legal and safe abortion, and women need better access to safe abortion and post abortion services. Otherwise, women in a great need of it, will continue to risk their lives by undergoing unsafe abortions [13]. In Ethiopia there is no research findings that showed change came as a result of abortion law implementation. Therefore this study was to assess knowledge on abortion law and associated factors among female regular undergraduate students of Ambo University.

## Methodology

### Study area and period

The institutional based cross sectional study was conducted in Ambo University which is located 114 km West of Addis Ababa the capital city of Ethiopian from March, 28-May, 18, 2018. Ambo University has 9 colleges/institutes, 38 academic departments and a total of 15,433 regular students of which 9675 were males and the rest 5758 were females [12]. A sample size of 795 was calculated by a single proportion formula, taking *P* = 50%, 95% confidence interval, α = 0.05 margin of error, d = 5% degree of precision, design effect of 2 and considering 10% non- response rate Stratified multistage sampling was implemented. In the first step the university stratified based on campus i’e main, technology and Guder campus. Then the campuses were stratified into colleges, departments and year of study These campus were further stratified in to as, colleges, departments and year of study and the study participants were calculated from each stratum using probability proportional to their size. Finally the study participants were selected using systematic random sampling technique using the student identification number as sampling frame.

The data were collected using structured questionnaire adapted from previous similar literature. The questionnaire includes socio-demographic and economic characteristics, questions that measure respondent’s knowledge on abortion law, source of acquiring information, and exposure of abortion and contraceptive related factors of the respondents. The question part that used to measure the respondent’s knowledge of abortion has skip pattern i’e if they answered ‘No’. The data entry was done with Epi Data version 3.1 and transported to Statistical package for social sciences (SPSS) version 21. Both descriptive statistics and logistic regression analysis were done. To have more candidate variables for multivariate logistic regression analysis a *p*-value of < 0.25 were considered as cut of point on bivariate logistic regression analysis. The abortion law knowledge part of the questionnaire contains eight items on yes or no score to which the respondents gave their response. The study participants level of knowledge on abortion law then measured using these eight abortion knowledge questions and respondents who score above or equal to the mean abortion knowledge was considered as having good knowledge and those who score less than the mean score was classified as poor knowledge for abortion law. Ethical approval to conduct the study was obtained from Ambo University, College of Medical and health sciences research and ethical review committee. The data was also collected after full informed verbal consent is obtained from the study participants.

## Result

All of the 795 undergraduate female students’ participated in the survey making the response rate of 100%. Majority of the respondents were in the age of between 18 and 24 with the mean ages of 21. About 62.5% of respondents’ age was above mean age while 37.4% were below mean age. About 47% of the study participants were protestant Christian followed by Orthodox (33.8%), Majority (86.4%) were single in marital status and 44.7% of them came from the rural areas of the country. Regarding current living arrangement, almost all respondents (91.4%) were living in campus and only 8.6% live out of the campus. The study participants distribution by year of study indicated that (29.5%) of them were first year, 61 30.8.0% second year, 25.0% third year, 11.1% fourth year, and 3.5% fifth year (Table 1).

**Table 1:** Socio-demographic characteristics of under graduate female students of Ambo University, West Shoa Zone, Ethiopia, 2018

Variables	N(%)
**Age**	
Below median age (21 years)	298 (37.4%)
Above median age (21 years)	497 (62.5%)
Religion	
Orthodox	269 (33.8%)
Muslim	103 (13%)
Protestant	374 (47%)
Wakefata	49 (6.2%)
**Marital status**	
Single	687 (86.4%)
Married	83 (10.4%)
Widowed	13 (1.6%)
Divorced	12 (1.5%)
**College or faculty**	
Natural science and computational	100 (12.6%)
medicine and health sciences	50 (6.3%)
Social science and Humanities	174 (21.9%)
Institute of cooperative	12 (1.5%)
College of business and Economics	40 (5%)
Institute of technology	260 (32.7%)
**Mother educational level**	
No formal education	244 (30.7%)
primary school	231 (29.1%)
High school	148 (18.6%)
College and above	172 (21.6%)
**Father educational level**	
No of formal education	135 (17.0%)
Primary school	234 (29.4%)
High school	178 (22.4%)
College and above	248 (31.2%)
**Previous Residence area**	
Urban	277 (34.8%)
Semi-urban	163 (20.5%)
**Current residence**	
In Campus	727 (91.4%)
Outside campus	68(8.6%)
Pocket money per month	
< 300	180 (22.6%)
Above 300	61(77.4%0
Estimated family income	
< 4500 or below	389 (48.9%)
Above 4500	406 (51.1%)

### Knowledge on abortion law

Regarding abortion law, 58.9% of the study participants hear about the existence of abortion legislation in Ethiopia of which the majority (55%) heard from clubs in school/University and followed by relatives / someone who had an abortion (48.1%). Among the respondents, 55.1% of them hear that the law in Ethiopia allows for legal abortion while 44.9% didn’t. Among those who knew that abortion is legal, 57.4% of them knew as abortion is permitted under all circumstances while 42.6% said abortion is only permitted under certain circumstances. Of the total respondents who reported abortion is legal under certain circumstances, 37.2% mentioned abortion is legal for pregnant mothers who have mental or physical disability followed by 36.0% for serious fetal deformity and 34.9% if pregnancy is as a results of rape, 33.3% if the pregnancy threaten the life of mother while the least 26.7% is if pregnancy is from incest.

In this study, 79% of the study participants were not knowledgeable about abortion law while 21% were knowledgeable (Table 2).

**Table 2:** Sources of information and knowledge on abortion legislation among female under graduate students of Ambo University, West Shoa Zone, Ethiopia 2018

Variables	N(%)
**Heard about abortion legislation**	
Yes	468 (58.9%)
No	327 (41.1%)
**Sources of information on abortion legislation**	
Received health education, on abortion legislation	180 (38.2%)
From TV/Radio	130 (27.8%)
From relatives / Someone who had an abortion	225 (48.1%)
From clubs in school/University	260 (55.6%)
The Current abortion law allow a women to have abortion	
Yes	258 (55.1%)
No	210(44.9%)
If yes,	
Under all circumstances	148 (57.4%)
Under some circumstances	148 (57.4%)
The current law on Abortion allows the woman on rape to run abortion	
Yes	90 (34.9%)
No	144 (55.8%)
The current law on Abortion allow the woman on incest to run abortion	
Yes	69 (26.7%)
No	163(63.2%)
The current law on Abortion allow the woman if the pregnancy threaten the life of mother.	
Yes	86(33.3%)
No	160(62%)
The current law on Abortion allow the woman if the mother has physically or mentally problem.	
Yes	96(37.2%)
No	142(55%)
Does the current law on Abortion allow the woman if serious fetal deformity	
Yes	93(36%)
No	133 (51.6%)
Over all abortion legislation knowledge	
Knowledgeable	98 (21%)
Not knowledgeable	370 (79%)

### Pregnancy, abortion and contraceptive utilization

From all respondents, 9.9% of them had a previous history of pregnancy and about 72.2% of those ever pregnant were reported that their pregnancy is not intended. Those who ever had pregnant, the majority had history of induced abortion of whom 21% of them aborted at health institution, 40.3% at traditional healers\ and 30% in their own house. Reasons were given why they had induced abortion. The majority, 40.3% of them reported to continue schooling and 2 of the girls reported for they had suffered rape. The methods used to induce abortion were roots and leaves 61.2%, plastics tube 18%, and oral drugs 9%. Among exposed to abortion 86.6% of the participants visited health facility due to problems related to induced abortion. Among all respondents, 81.3% of them heard about emergency contraceptive of which nearly half of the respondents 48.7% described they are currently using contraceptives among which condoms are the highest percentage (32.0%) (Table 3).

**Table 3:** Pregnancy and Abortion related frequency distribution among female under graduate students of Ambo University, West Shoa Zone,Ethiopia, 2018

Variables	N(%)
Ever been pregnant	
Yes	79 (9.9%)
No	716 (90.1%)
Intended pregnancy	
Yes	22 (17.8%)
No	57 (72.2%)
Reason for not intended	
Faced Physical violence	27 (47.4%)
Faced Sexual violence	26 (45.6%)
Rape	4 (7%)
Ever undertaken abortion	
Yes	67 (85%)
No	12 (15.2%)
The place where induced abortion done.	
Health facility	14 (21%)
Abortionists houses	27 (40.3%)
Your house	20 (30%)
Others	6 (9%)
Method which used for induced abortion	
Plastic	12 (18%)
Oral drugs	6 (9%)
Roots and leaves	41 (61.2%)
currently using contraceptive method	
Yes	387 (48.7%)
No	408 (50.9%)
Method of contraceptive used	
Pills	55 (6.9%)
Condom	255 (32%)
Inject able	70 (8.8%)
Other	34 (4.3%)
Ever heard about emergency contraceptive	
Yes	646 (81.3%)
No	149 (18.7%)

### Predictors of abortion law knowledge

In this study all socio-demographic variables did not have an association with kowlege of abortion law. Study participants who were involved in the health club in the university, knew the place where induced abortion can be done and received health education on the abortion law were identified as the predictor of abortion law knowledge. Among those who know that abortion is legal under certain circumstances, 50.9% were being member of health club in school. The odds of having good knowledge of abortion law are 2.09 times more likely among students who were member of health club when compared with those who were not a member of health club (AOR = 2.09, 95% CI: (1.256–3.484)). Students knowing the place where abortion can be conducted have 3.12 times the odds of having good knowledge of abortion law than those who did not know where abortion can be done (AOR = 3.12, 95% CI: (1.854–5.239)). In addition, students received health educations on the abortion law are 7.38 times more likely to have good knowledge of Abortion Law compared to those who did not get health education on Abortion law (AOR = 7.38, 95% CI: (1.118–48.761)) (Table 4).

**Table 4:** Binary and multiple Logistic regression analysis factors associated with knowledge of Abortion legislation, among under graduate female students of Ambo University, West Shoa Zone, Ethiopia, 2018

Variables	Knowledge of legal abortion		COR (95% C. I)	AOR(95% C.I)	*p*-value
	Poor (N)	Good (N)			
Year of study					
**Year I**	140	30	1	1	
**Year II**	105	22	.98(.534–1.792)	1.16(.598–2.250)	.661
**Year III**	78	25	1.496(.822–2.722	1.55(.811–2.965)	.184
**Year IV**	39	14	1.675(.810–3.465)	2.00(.914–4.373)	.083
**Year V**	8	7	4.083 (1.375–12.125)	3.20(.950–10.772)	.061
Know the place where safely induced abortion performed					
**Yes**	137	66	3.51 (2.188–5.624)	3.12 (1.854–5.239)	0.000^a^
**No**	233	32	1	1	
Know some-one who had Abortion					
Yes	118	46	2.25 (1.429–3.542)	1.46(.880–2.426)	.143
No	252	52	1	1	
Ever visited health institution					
Yes	214	71	1.92 (1.176–3.125)	1.59(.927–2.725)	.092
No	156	27	1	1	
Received health education on abortion legislation					
Yes	117	43	1.69 (1.072–2.665)	7.38 (1.118–48.761)	0.038 ^a^
No	253	55	1	1	
Being member of health club in schools					
Yes	145	58	1.89 (1.201–2.972)	2.09 (1.256–3.484)	0.01 ^a^
No	225	40	1	1	
Currently using contraceptive					
Yes	164	59	1.87 (1.189–2.948)	1.44(.858–2.403)	0.169
No	203	39	1	1	

^a^indicate factors that showed association with outcome variable

*Statistically significant at 95% confidence interval.at *p*-value 0.05

## Discussion

The study revealed that 21% of the respondents have good knowledge on abortion legislation while 79% have poor knowledge. This result is higher than the study reported from Ghana, which only 3% of women reported as having knowledge on abortion legalization [15]. This difference is probably due to the difference in study design in which the study conducted in Ghana is a community-based survey which included all reproductive age women while this study is institutional based.

On the other hand the finding of this study is much lower compared to a study from South Africa, which revealed that 68% of the study participants knew that abortion is legal [10]. This difference is probably due to the difference in circumstance of the existing abortion law in South Africa is more applied better than the study setting. In addition the difference is probable due to the study design in which the study in South Africa is community based which involved reproductive age groups. The finding of this study is also lower when compared to the result of the study done at Harari region Eastern Ethiopia in which 35.7% of female students reported as having good awareness to legalization of safe abortion [16]. This difference could be due to poor information dissemination to the target population about their reproductive health issues in the study setting .

This finding is also lower than the studies from Ethiopia among reproductive age women at Yirgachefe town, where (48.9%) of them know abortion is legal in Ethiopia under certain circumstances [17]. The difference might be due to the socio-cultural difference between the two populations. Since this study was conducted in university, the respondents came from different corners of the country having different cultural, religious and ethnic backgrounds. But the study from Yirgachefie includes all women in the reproductive age group who have similar backgrounds.

The result of this finding is also lower when compared to study done among preparatory students at Dabat District of Ahmara region, where 62.8% students know that the law in Ethiopia allows safe and legal Abortion under certain circumstances [18]. This difference might be due to study setting difference and health facility coverage at Dabat district which works with stakeholder like Engender to expand safe abortion service and gives information about Abortion legality.

The result of our study also shows that nearly 79% of students did not know that abortion is allowed under certain circumstances in Ethiopia. This is somewhat close to the finding of the study done in Wolayita Sodo University (61%) of women reported that they don’t know abortion is legal [19]. This is due to similar socioeconomic and educational status of the study population, similar study setting. This result is lower than study done at Ghana which shows 92% of the women are not aware that abortion was permitted by law under certain circumstances in Ghana [20]. This difference is may be due to population difference, study setting and focus of MOH for Abortion service and education between two countries and socioeconomic difference.

In addition, this study also shows that 79(9.9%) of the students had a previous history of pregnancy and 67(85%) had history induced abortion. The magnitude of induced abortion in this study is much higher than the related study from Ethiopia, Ahmara region, Dabat district 10 (43.5%) [21]. this difference is may be because of different study population and place of residence since the preparatory students in Dabat District are living with their family and monitored. About 40.3% of the abortions reported in this study were carried out at the illegal and unlicensed abortion houses. That means nearly half of the induced abortions reported in this study were unsafe. This implies that unsafe abortion is still a major maternal health problem affecting young female students. This is much higher with the finding reported from Arba Minch University students, 17% of the abortions conducted were reported to be unsafe abortion [22]. Knowledge about abortion law has significant implications for accessing legal and safe induced abortion services. Larger proportions of the abortions were not conducted through legal and safe procedures while abortion is legal under certain circumstances. Another finding of this study is respondents’ use of roots and leaves to induce abortion, this might cause further complications. One possible reason that leads women to practice unsafe abortion might be the lack of knowledge of the abortion law. This is strongly supported by various studies [18, 22].

This study showed that there is a significant association between knowledge of abortion law and received health educations on the abortion legislation, knowing places where abortion is conducted and Being member of health club in school. Students received health educations on the abortion legislation are 7.38times more likely to have good knowledge of abortion legislation. The possible explanation might be due to their better Awareness they got from health education regarding revised abortion law, increased exposure to maternal health and abortion-related information.

Respondents’ awareness of where safe abortion can be performed is significantly associated with knowledge of abortion legislation. Students knowing the place where abortion can be conducted have 3.116 times the odds of having good knowledge of abortion legislation than those who did not know where abortion can be done. This finding is in line with findings from Dabat District among female preparatory students [19]. This might be due to their improved information exposure and health facility access. Students who were member of health club are 2.092 times more likely to have knowledge of Abortion legislation when compared with those who were not a member of health club. This might be due to the reason that those who were member of health club can expose to health information related to different issues including Abortion.

## Conclusion

Overall, this finding indicates that Ambo University female regular students had limited knowledge on the existence of legal abortion in Ethiopia. Participants who received health education about abortion law, who knew where abortion can safely be performed and those who involved in health club were found to be predictors of abortion law knowledge Therefore ministry of health had better to work and encourage stakeholders on dissemination of information regarding the existing abortion law.

### Strength of the study

One of the strength of this study was able to achieve 100% response rate. This could be true as the data were collected by interview methods and the interview was done by well trained professionals.

### Limitation of the study

The data from this study may not represent all reproductive age groups because the study respondents were limited to only university female students.

In addition, there is lack of data and literature for the comparative analysis of the result.

In addition to these the limitation of this study was the smallness of the sample size.

## Data Availability

The datasets during and/or analyzed during the current study are available from the corresponding author on reasonable request.

## Abbreviations

AOR: Adjusted odds ratio
CAC: Compressive abortion care
CI: Confidence interval
COR: Crude odds ratio
FMOH: Federal Minister of Health
GA: Gestation age
ICPD: International Conference on Population and Development
SDG: Sustainable development goal
WHO: World Health Organization
